# Cisplatin chemotherapy (without erythropoietin) and risk of life-threatening thromboembolic events in carcinoma of the uterine cervix: the tip of the iceberg? A review of the literature

**DOI:** 10.1186/1748-717X-1-14

**Published:** 2006-05-05

**Authors:** Jon C Anders, Perry W Grigsby, Anurag K Singh

**Affiliations:** 1 Radiation Oncology Associates, Albuquerque NM 87109, USA; 2 Washington University School of Medicine, Department of Radiation Oncology, St. Louis MO 63110, USA; 3 National Cancer Institute, Radiation Oncology Branch, Bethesda MD 20892, USA

## Abstract

**Background:**

The risk of severe cardiovascular toxicity, specifically thromboembolic events (TE), in patients with cervical cancer receiving concurrent irradiation and cisplatin chemotherapy is reported to be less than 1% in several large prospective trials. However, the anecdotal risk appears to be far higher.

**Results and discussion:**

A review of several prospective trials demonstrates no treatment related grade 4 cardiovascular toxicities and only two grade 5 toxicities in 1424 (0.1%) collective patients. A recent publication and our own unpublished experience finds 6 of 128 (4.7%) patients developed grade 4 to 5 cardiovascular (thrombosis/embolism) toxicity. The differenc in incidence of severe or life threatening cardiovascular toxicity of 0.1 versus 4.7% is highly statistically significant (p < 0.00001.)

**Conclusion:**

This dramatic difference in incidence of cardiovascular toxicity raises the possibility that cardiovascular toxicities were inadequately reported on the listed prospective trials. For those patients enrolled in prospective trials, we suggest that thromboses should be diligently documented and reported. Only after the true incidence of thromboses is established can we implement appropriate levels of early screening and intervention that may prevent life threatening complications.

## Background

A retrospective, case control study of 147 with carcinoma of the cervix or vagina treated with chemoradiotherapy with or without erythropoietin showed a 23 versus 3% incidence of TE. [1] Such recent findings of an elevated risk of cardiovascular toxicity, specifically thromboembolic events (TE), in patients receiving concurrent irradiation, cisplatin chemotherapy and erythropoietin have spurred interest in the true incidence of TE in patients receiving concurrent irradiation and cisplatin chemotherapy in the absence of erythropoietin.

The use of cisplatin, either alone or in combination with other chemotherapeutic agents, has become the standard of care for the treatment of various solid tumors. Specifically, the routine use of cisplatin in the treatment of cancers of the uterine cervix has been cemented with the publication of several recent prospective randomized trials [2-8].

When reporting the results of these prospective trials, the scoring of treatment related toxicity is site specific. For example, TE are scored as cardiovascular toxicity and graded from 1 to 5 on the RTOG scale (Table 1). However, these trials often do not specify the incidence and severity of treatment related cardiovascular (thrombotic) toxicities. In fact, of the trials shown in Table 2, incidence of TE were only specifically reported in the study by Malfetano et al [4].

**Table 1:** RTOG Cardiovascular (Thrombosis/Embolism) Toxicity Scoring

Grade	
1	--------------
2	DVT not requiring anticoagulation
3	DVT requiring anticoagulation
4	Pulmonary embolism from thromboses
5	Death

**Table 2:** Incidence of thromboembolic toxicity in prospective studies using cisplatin

**Trial**	**Chemotherapy**	**XRT**	**Toxicity**	**No. CTX Pts**
Keys et al. [5] (cervix)	Cis 40 mg/m^2 ^q wk X 6 concurrent	75 Gy to pt A	0 deaths 1 Grade 3&1 Grade 4 CVT (NOS)	183
Benedetti et al. [3] (cervix)	Cis 40–80 mg/m^2 ^q wk X 6–8 concurrent	45–50 Gy WP 20–30 Gy Low Dose Rate	0 deaths	201
Morris et al. [2] (cervix)	Cis 75 mg/m^2 ^and 5-FU 4000 mg/m^2 ^q wk X 3 concurrent	85 Gy to pt A	1 death (NOS)	193
Pearcey et al. [6] (cervix)	Cis 40 mg/m^2 ^q wk X 5 concurrent	80 Gy to pt A	1 death (SBP) 3 Grade 3 CVT (NOS)	127
Peters et al. [7] (cervix)	Cis 70 mg/m^2 ^and 5-FU 1000 mg/m^2 ^q wk X 4 concurrent	4930 WP @ 170 cGy/day	1 death (Bilateral ureteral obstruction)	127
Rose et al. [8] (cervix)	Cis 40 mg/m^2 ^q wk X 6 concurrent or Cis/5-FU/Hydroxyurea or Hydroxyurea	80 Gy to pt A	0 deaths 2 Grade 3 CVT (NOS), with 3 drug regimen	526
Malfetano et al. [4] (cervix)	Cis 1 mg/Kg q wk with XRT	45 Gy PA, WP 4–5000 cGy and 3–4000 cGy Low Dose Rate	2 Grade 5 CVT (from PE)	67

NOS = Not otherwise specified CVT = Cardiovascular Toxicity SBP: Small Bowel Perforation Cis = Cisplatin 5FU = 5 Fluorouracil WP = Whole Pelvis Gy = Gray

## Results and discussion

A review of these prospective trials demonstrates no treatment related grade 4 cardiovascular toxicities and only two grade 5 toxicities (Table 2) in 1424 collective patients. According to the literature then, formation of severe or life threatening thromboses associated with cisplatin chemotherapy, in the absence of erythropoietin, is an exceedingly rare event.

The data in table 3, however, belies such rarity. A recent publication and our own unpublished experience yields (Table 3) 6 cases of grade 4 to 5 cardiovascular (thrombosis/embolism) toxicity in a cohort of 128 patients. The incidence of severe or life threatening cardiovascular toxicity in tables 2 and 3 was 0.1 versus 4.7%, p < 0.00001.

**Table 3:** Incidence of thromboembolic toxicity in recent retrospective cohorts using cisplatin

**Trial**	**Chemotherapy**	**XRT**	**Toxicity**	**No. CTX Pts**
Jacobsen et al [9] (cervix)	Cis 40 mg/m^2 ^q wk X 6 concurrent	85 Gy to pt A	1 Grade 5 3 Grade 4 CVT	48
Mallincrodt (unpublished) (cervix)	Cis 40 mg/m^2 ^q wk X 6 concurrent	85 Gy to pt A	1 Grade 5 1 Grade 4	80

NOS = Not otherwise specified CVT = Cardiovascular Toxicity SBP: Small Bowel Perforation Cis = Cisplatin 5FU = 5 Fluorouracil WP = Whole Pelvis Gy = Gray

Jacobson et al found a 16.7% incidence of TE 48 patients treated with definitive chemoradiation for cervical cancer. Four of these 48 patients developed grade 4–5 TE. [9] Of these 4 events, there were 3 grade 4 toxicities and 1 grade 5 toxicity. This is consistent with our unpublished institutional experience with 1 grade 4 and 1 grade 5 toxicity in a cohort of approximately 80 patients with pelvic malignancies treated with radiation and cisplatin chemotherapy, without erythropoietin.

The development of thromboembolic disease is dependent upon the relationship between the factors of Virchow's triad: stasis, hypercoagulability, and venous injury. As first described by Trouseau in the nineteenth century, and supported by modern publications, some patients with malignancy are hypercoagulable and do develop thromboses. [10,11] Simply from their malignancy, in the absence of chemotherapy, one might expect more than 2 reported cases of severe thrombotic events out of the 1424 patients described in Table 2.

In addition to the increase of thromboses as a result of malignancy, a review of chemotherapy associated vascular toxicity suggests that chemotherapeutic agents may increase the risk of thromboses by damaging vessel walls or producing changes in the clotting cascade [12]. Feffer et al. [13] reported that patients receiving chemotherapy for breast cancer showed a statistically significant reduction of functional protein C levels that returned to normal upon completion of therapy. Icli and associates [14] suggested that this severe vascular toxicity may be related to hypomagnesaemia, autonomic dysfunction, alteration in platelet aggregation, elevated plasma von Willebrand factor and hypercholesterolemia. Echoing these findings, several recent publications suggest that the incidence of venous thrombosis is further elevated in those patients receiving chemotherapy. [15-17]

Through vascular injury and possible alterations in the clotting cascade, chemotherapy agents such as cisplatin have the ability to affect coagulability and cause vascular injury, two aspects of Virchow's triad. Thus, though unsupported by the data from the trials summarized in Table 2, there is a theoretical basis to support the increased incidence of TE reported in table 3.

Venous stasis is well documented to cause thromboembolic events. The 6 events documented in Table 3 occurred in cervix cancer patients. It might be hypothesized that cervix cancer patients undergoing prolonged, in-patient brachytherapy procedures may be at a high risk for the development of DVT. Several retrospective studies of the perioperative morbidity and mortality of gynecologic brachytherapy have been performed [18-21]. These studies were performed in patients not receiving concurrent cisplatin chemotherapy and no excess risk of TE was described.

It remains possible that venous stasis during brachytherapy interacts with cisplatin to produce higher incidence of thromboembolic events. However, only 1 of the 4 grade 4–5 TE described by Jacobson was associated with brachytherapy. Moreover, 6 of the 7 trials listed in Table 2 were performed in cervix cancer patients who underwent brachytherapy. Therefore, if the events in table 3 solely are due to venous stasis during brachytherapy interacting with cisplatin to produce higher incidence of thromboembolic events, then the similar patients from the randomized trials in Table 2 should have had a similar rather than a statistically significant difference (p < 0.00001) in incidence of TE.

## Conclusion

Combining the results of a recent publication and our own experience, we note 6 cases of grade 4 or 5 TE in patients receiving cisplatin and concurrent irradiation without erythropoietin for malignant disease including two deaths from thromboses (Table 3). Such an incidence is consistent with the known pro-thrombotic effects of malignancy and chemotherapy. However, data from prospective trials (Table 2) reported only 2 of 1424 having grade 4 or 5 TE. The dramatic difference in incidence of cardiovascular toxicity between Tables 2 and 3, raises the possibility that cardiovascular toxicities (specifically thrombosis, embolism) were inadequately reported on the listed prospective trials.

For those patients enrolled in prospective trials, we suggest that thromboses should be diligently documented and reported. Only after the true incidence of thromboses is established can we better evaluate the therapeutic ratio of cisplatin therapy with or without novel agents such as erythropoeitin. Also, this will allow the implementation of appropriate levels of early screening and intervention that may prevent life threatening complications.
